# A hydroxyapatite-based synthetic bone graft reduces intervertebral motion at 12 months, as measured by CT-RSA, in a porcine model of ACDF

**DOI:** 10.1016/j.bas.2026.106284

**Published:** 2026-08-13

**Authors:** Maria Östman, Peter Försth, Olof Sandberg, Odd Höglund, Patricia Hedenqvist, Håkan Engqvist, Michael Pujari-Palmer, Franck Forterre

**Affiliations:** a Department of Clinical Veterinary Medicine, Division of Small Animal Surgery, Vetsuisse Faculty, University of Bern, Bern, Switzerland; b Department of Surgical Sciences, Division of Orthopedics, Uppsala University, Uppsala, Sweden; c Sectra AB, Sweden; d Department of Clinical Sciences, Swedish University of Agricultural Sciences, Uppsala, Sweden; e Department of Materials Science and Engineering, Division of Applied Materials Science, Uppsala University, Uppsala, Sweden

**Keywords:** CTMA, Pseudarthrosis, Spinal fusion, Pig, Computed tomography, Anterior cervical discectomy and fusion

## Abstract

**Introduction:**

Pseudarthrosis remains a significant complication after anterior cervical discectomy and fusion (ACDF), often causing revision surgery. Synthetic bone grafts (SBGs) offer an alternative to autografts, but objective methods for assessing intervertebral motion (IVM) are needed. Computed tomography-based radiostereometric analysis (CT-RSA) enables precise measurement of cervical IVM.

**Research question:**

Does a hydroxyapatite-based SBG reduce cervical IVM compared with control in a porcine ACDF model measured by CT-RSA?

**Material and methods:**

Six adult female mini-pigs underwent two-level cervical anterior discectomy. One defect received a hydroxyapatite-based SBG, and the other served as an empty control. No instrumentation was used. The primary outcome was induced total rotation (TR) between vertebrae during flexion-extension CT at 9 and 12 months, compared using the Wilcoxon matched-pairs signed-rank test. Functional fusion was defined as TR < 1° and compared using exact McNemar test. Median (range) values are presented.

**Results:**

At 12 months, median IVM TR was significantly lower at SBG than Control levels (0.2° [0.07–0.5] vs. 1.5° [0.2–5.0], p = 0.031). At 9 months, TR was 0.3° (0.04–1.0) and 0.8° (0.1–2.7), respectively (p = 0.063). Functional fusion rates were higher at SBG levels (5/6 and 6/6 at 9 and 12 months) than at Control levels (3/6 at both time points), although the differences were not statistically significant.

**Discussion and conclusion:**

The hydroxyapatite-based SBG was associated with lower IVM at 12 months in this porcine ACDF model. CT-RSA provided an objective method for assessing cervical segmental stability and may be useful for evaluating functional spinal fusion in preclinical studies.

## Highlights

• CT-RSA objectively quantified cervical intervertebral motion in vivo.
• Hydroxyapatite graft was associated with lower intervertebral motion at 12 months.
• CT-RSA may aid assessment of functional spinal fusion in preclinical models.

## 1 Introduction

Anterior cervical discectomy and fusion (ACDF) is the gold-standard surgical technique for treating degenerative cervical spinal diseases in human medicine (Cloward, 1958; Pavlov, 2003). However, following ACDF procedures, pseudarthrosis is a significant complication with an incidence ranging from 7.7% to 12.4% (Tavanaei et al., 2025); and is associated with persistent neck pain, vertebral instability, radiculopathy, and myelopathy (Zuckerman and Devin, 2022). Pseudarthrosis after ACDF is a leading cause of revision surgery associated with further reduced quality of life (Pennington et al., 2020). Reoperating rates are reported to range from 0.58% to 11.1% (Noordhoek et al., 2019; Shousha et al., 2019; Wewel et al., 2019). Autologous bone grafts are considered the gold standard for promoting bony fusion and preventing pseudarthrosis. However, synthetic bone grafts (SBGs), such as calcium phosphate-based materials, are frequently used as alternatives because of their ease of application during surgery, shorter operative times, and ability to eliminate donor site morbidity (Habraken et al., 2016; Gupta et al., 2015; Almaiman et al., 2013). An accurate diagnosis is essential for the effective treatment and management of pseudarthrosis. However, the diagnosis is complicated because not all patients with pseudarthrosis are symptomatic, and not all patients require revision surgery (Pennington et al., 2020). To diagnose pseudarthrosis after cervical spine surgery, clinicians have traditionally used a) subjective assessment of bony bridging and detection of any radiolucent line on cervical X-ray along with b) assessment of cervical intervertebral motion (IVM) on flexion‒extension X-rays using <2° as a threshold defining fusion (Noordhoek et al., 2019), although some authors have suggested a cutoff of <1° (Hipp et al., 2024). Accurate measurement and interpretation of IVM are essential for diagnosing patients with symptomatic pseudarthrosis, however clinically practical and reliable diagnostic tools are lacking (Benson et al., 2022; Peters et al., 2019; Rhee et al., 2015). Computed tomography-based radiostereometric analysis (CT-RSA) is a promising tool for evaluating intervertebral cervical micromotion (Sandberg et al., 2023; Svedmark et al., 2011). CT-RSA can detect relative micromotion between vertebrae with high accuracy by comparing two sets of images, one in flexion and the other in extension, using imaging stabilization (Hutchins et al., 2024). The precision of CT-RSA has been shown to be comparable or superior to that of radiostereometric analysis (RSA) in implant migration studies and ranges between 0.13° and 0.31° for total rotation and 0.10-0.16 mm for translation (Sandberg et al., 2023; Brodén et al., 2021; Engseth et al., 2023; Östman et al., 2021).

Previous research on a similar SBG in a porcine ACDF model focused on morphological fusion via subjective CT assessments (Östman et al., 2024) and preliminarily demonstrated the utility of the CT-RSA for *ex vivo* functional fusion assessment (Östman et al., 2021). The current study aimed to evaluate the effect of hydroxyapatite-based SBG on cervical IVM compared with the control in a porcine model of ACDF using CT-RSA. It was hypothesized that the SBG would decrease IVM compared to the control, measured by CT-RSA, in this porcine model of ACDF.

## 2 Methods

### 2.1 Study design and animal model

The present study was approved by the Animal Ethics Board in Uppsala, Sweden (protocol: 08751-2019) and adhered to Good Laboratory Practices at the University of Agricultural Sciences in Uppsala, Sweden, between March 2022 and June 2023, in compliance with Swedish national legislation for the care and use of laboratory animals and “Principles of laboratory animal care” revised, 1985.

Six individually indoor-housed adult female Danish Göttingen mini-pigs (N = 6; Ellegaard Göttingen Minipigs) were included in the study, with demographics as described in Online Resource 1. Anterior partial discectomies were performed to create intervertebral defects at both an upper and lower level of the cervical vertebral column in each animal. One defect was randomly allocated to receive SBG, with the remaining defect serving as an empty internal negative control, randomization as described in Online Resource 2. The primary outcome was IVM, which was measured as the total rotation (TR) between two vertebrae in degrees using flexion-extension CT scans at 9- and 12-months postoperatively. The secondary imaging outcomes included assessments of TR from C2 to C7, changes in TR before and after surgery, IVM in Adjacent segments, and the functional fusion rate. Functional fusion was classified as fused (<1° TR) or not fused (≥1° TR).

### 2.2 Calcium phosphate ceramic preparation

The ceramic was prepared as a putty consisting of 40% ceramic material and 60% of the carrier. The carrier was composed of ascorbic acid and carboxymethylcellulose. By weight, the ceramic material was composed of 90-95% hydroxyapatite, 1-4% calcium pyrophosphate, 1-5% alpha-tricalcium phosphate, and less than 5% monetite. This composition was verified by powder XRD in a previous study. The ceramic material was synthesized according to a previously described method (Östman et al., 2024).

### 2.3 Surgical procedure

A ventral median approach was used to access each cervical vertebral column at two different levels, allowing for the exposure of the intervertebral spaces at one upper level, C2/3 or C3/4, and one lower level, C4/5 or C5/6, while maintaining the integrity of the intermediate level. One level randomly received SBG, and the other served as a control, resulting in an equal distribution of SBG and Control levels between the upper and lower cervical vertebral column. All surgical procedures were conducted under general anesthesia, with analgesia provided during and after the operation according to the protocol described in Online Resource 3. The cervical intervertebral discs were partially excised, creating partial anterior vertebral body defects of similar volume (mean [SD]: SBG 710 [202] mm^3^; Control 596.7 [130] mm^3^). Portions of the anterior disc tissue, along with parts of the end plates and the vertebral bodies, were resected. The dorsal longitudinal ligament was left intact. Following resection, one defect was filled with SBG material, and the other was left empty. No vertebral fixation was used, and standard closure techniques were employed. All procedures were performed by the same board-certified veterinary surgeon. The animals were monitored by veterinarians according to the postoperative protocol in Online Resource 4 and by video surveillance as part of another study for 7 days. No postoperative activity or motion restriction was imposed.

### 2.4 CT-RSA analysis

**Fig. 1:**
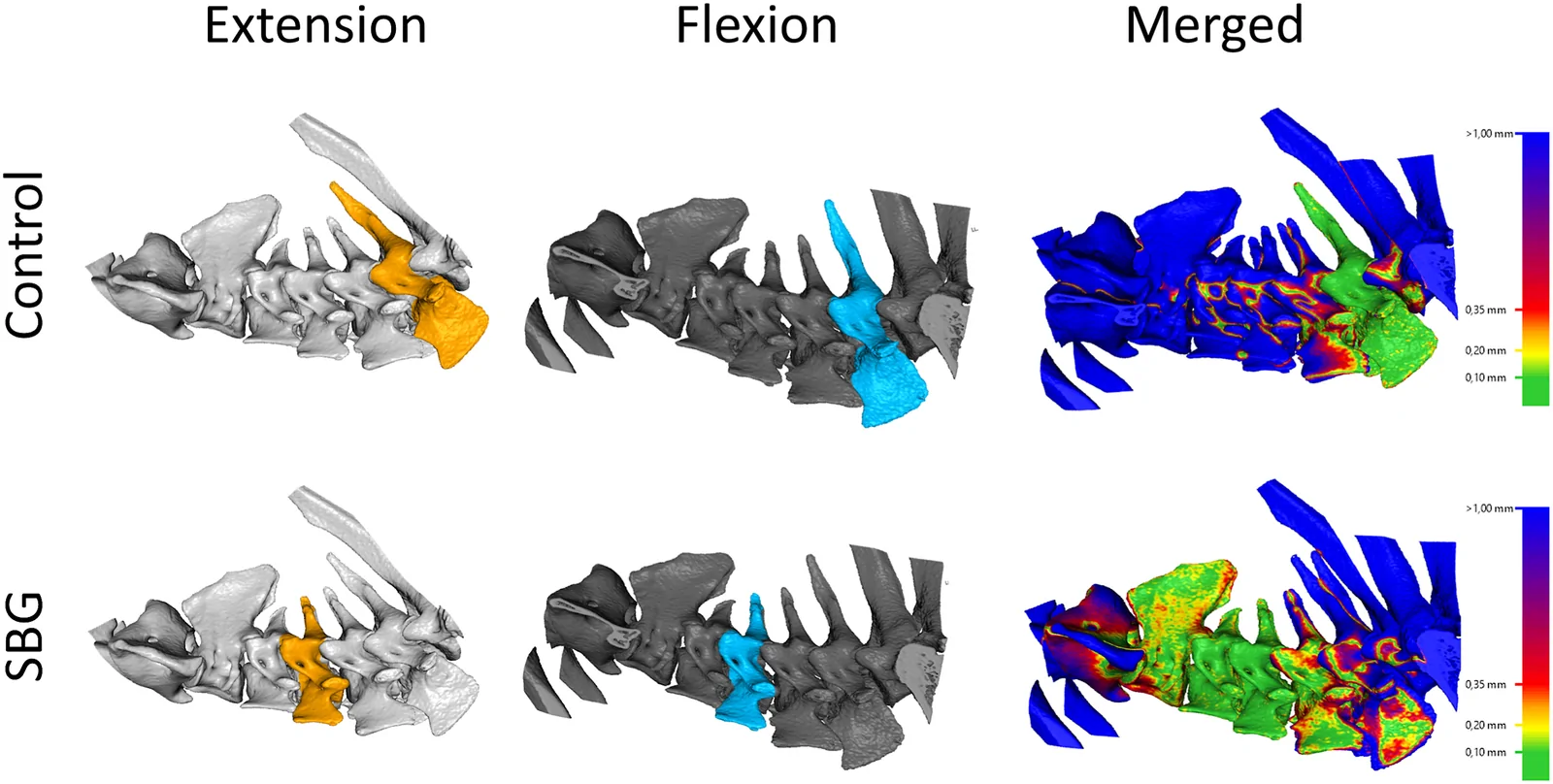
Representative CT-RSA analysis of intervertebral motion (IVM) in the Control and SBG-treated levels. Three-dimensional computed tomography volumes are shown during extension (left), flexion (middle), and the merged position (right) The rigid body is highlighted in orange during extension and blue during the flexion, the remaining vertebral structures are shown in grey. The merged reconstructions visualize and quantify the degree of induced motion between the rigid body and the vertebra cranial to it. The colour scale represent the magnitude of IVM in mm where green represents low motion and blue high motion.

All animals underwent flexion-extension CT examinations prior to surgery and at 9- and 12-months postoperatively, according to the CT protocol in Online Resource 5. The cervical vertebral column was scanned using a Siemens Somatom Definition AS 64 CT scanner at an isotropic resolution of 0.6 mm. For 3D multiplanar reconstructions, a 0.6 mm slice thickness and a B70s kernel were used as part of the Spine Helical series. The resulting voxel size was approximately 0.25 × 0.25 × 0.6 mm, with slight variations depending on the field of view and acquisition parameters. Blinded image postprocessing volume registration was conducted using the CT-RSA tool (CTMA, Sectra, Linköping, Sweden). Paired CT volumes were registered to measure the induced TR between the vertebrae. Using imaging stabilization, one vertebral body was designated as the rigid body, allowing for the measurement of movement in the adjacent cranial vertebra, designated as the moving body, using flexion and extension images (Fig. 1 and video in Online Resource 6). The Adjacent level was defined as the level immediately below the operated level that demonstrated the least IVM at 12 months. The measurement points for which induced displacements were reported included the top of the spinous process, anterior midsagittal vertebral body, and center of the facet joints on both the left and right sides. A set coordinate system was used for each analysis (according to the CT-RSA protocol in Online Resource 7). The change in TR was calculated as TR before surgery minus TR after surgery at 9- and 12-months.

### 2.5 Statistical analyses

Statistical analyses were performed using GraphPad Prism 10 (GraphPad Software, Boston, Massachusetts, USA) and R (R Foundation for Statistical Computing, Vienna, Austria). Medians with maximum and minimum values and means with standard deviations (SDs) were reported. Because each animal received both a Control-and a SBG level, each animal served as its own internal control. Comparisons between groups (Control vs. SBG, Control vs. Adjacent, and SBG vs. Adjacent) were performed separately at each time point using the Wilcoxon matched-pairs signed-rank test. Hodges–Lehmann estimates with exact confidence intervals were calculated to quantify the paired median differences and reported as measures of effect size. Fusion rates were compared using the exact McNemar test. No adjustments were made for multiple comparisons. Statistical significance was set at p < 0.05. According to the American Society for Testing and Materials (ASTM) F2884-21, a sample size of six to eight animals is sufficient for proof-of-concept. The raw data supporting this study are available in Online Resource 8.

## 3 Results

### 3.1 Total flexion-extension of the cervical vertebral column

**Fig. 2:**
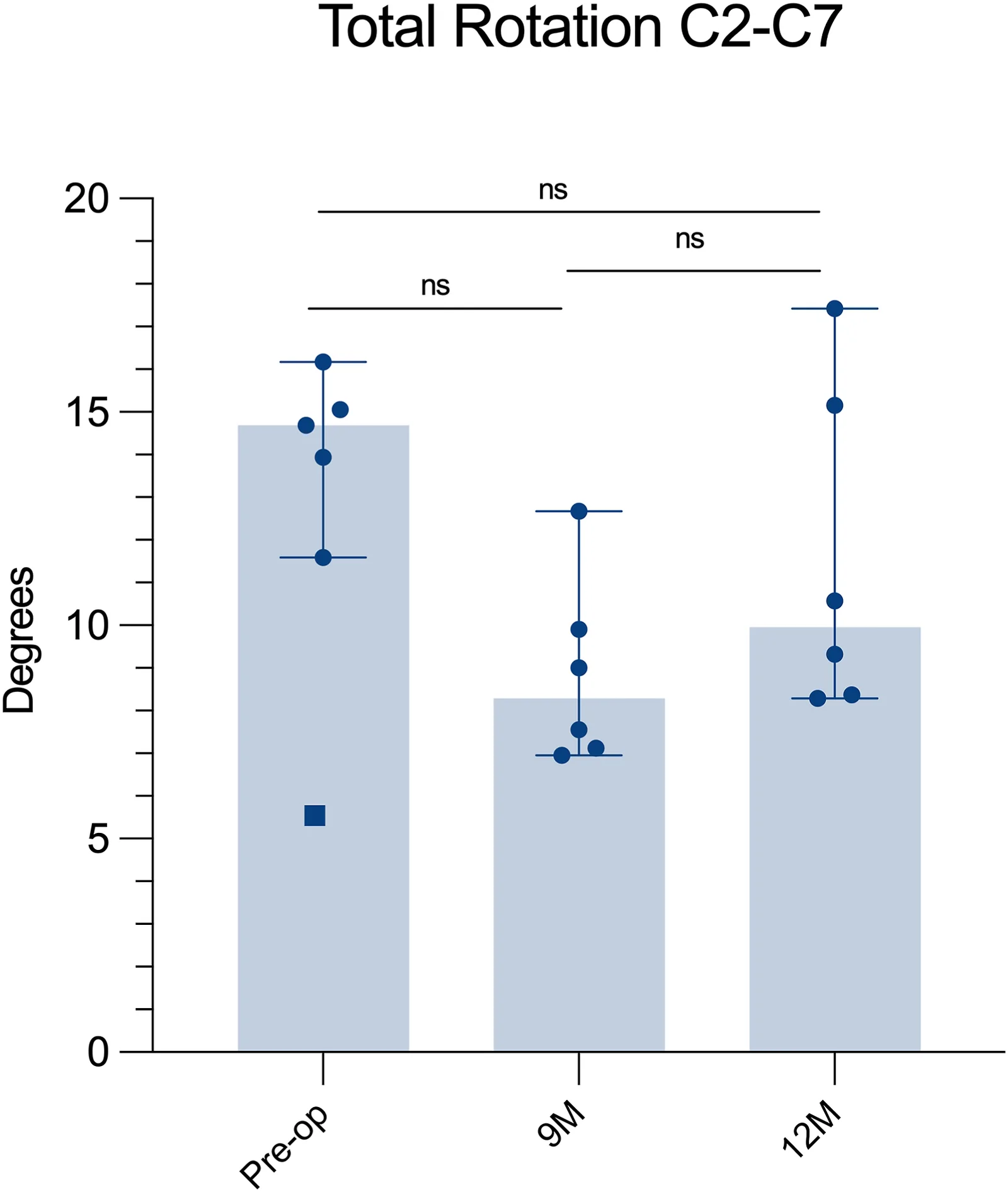
Median TR of flexion-extension from C2 to C7 with individual values plotted in the graph. No significant differences were observed in the total bending between time points, with or without the outlier marked as a square. The Wilcoxon matched-pairs signed-rank test was used for comparison. Statistical significance was set at p < 0.05. M = months after surgery.

The TR of flexion-extension from C2 to C7 was assessed at all time points to ensure adequate provocation of the vertebral column. One animal showed a notable deviation from the others before surgery, with a total bending of 5.3° (marked square in Fig. 2), compared with the remaining animals, which ranged between 11.6° and 16.2°. This animal was excluded from all preoperative IVM analyses. In contrast, the total C2–C7 motion of this animal at the postoperative examinations was comparable to that of the remaining animals (9 M: 9.0° vs. 7.0–12.7°; 12 M: 8.3° vs. 8.4–17.4°); therefore, it was retained in the primary analyses. No significant difference in the median TR of flexion-extension from C2 to C7 was detected between any timepoint with or without the outlier (pre-op N = 5: 14.7 [11.6–16.2]; pre-op N = 6: 14.3 [5.3–16.2]; 9 M post-op: 8.3 [6.9–12.7]; 12 M post-op: 9.9 (Fig. 2).

### 3.2 Intervertebral motion

**Fig. 3:**
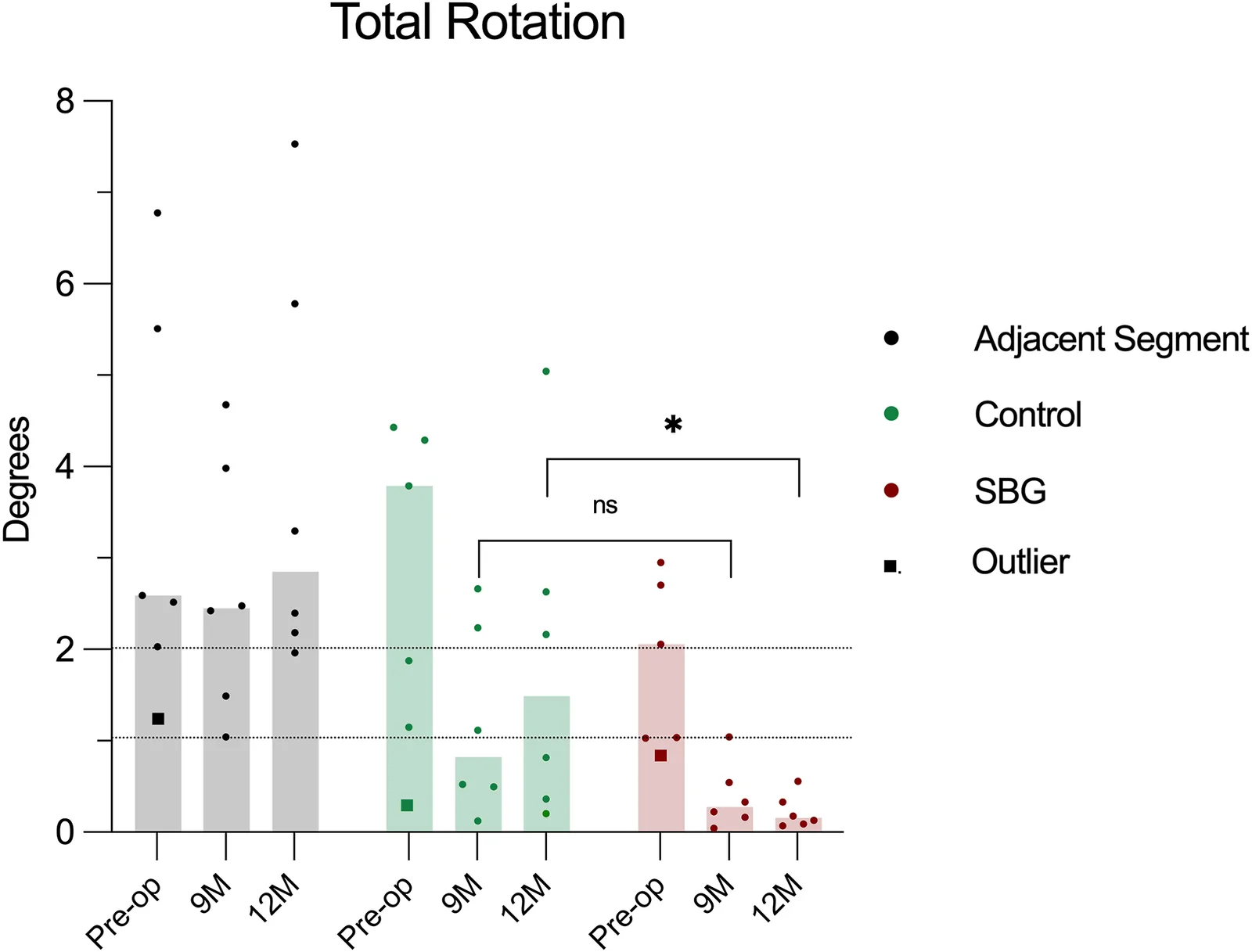
Median intervertebral motion (IVM) measured as total rotation in degrees before surgery (pre-op; N = 5), at 9-months (9 M; N = 6) and at 12-months (12 M; N = 6) postoperatively. Outlier was excluded from all analyses preoperatively, marked as a square. * Statistically significant (p < 0.05), SBG, synthetic bone graft. Dashed horizontal lines indicate the 1° and 2° IVM thresholds, respectively.

The median IVM TR was significantly lower at the SBG levels than at the Control levels 12 months postoperatively (*p* = 0.031; Table 1 and Fig. 3). At 9 months, there was no difference in the median IVM TR between the SBG and Control levels (*p* = 0.063; Table 1 and Fig. 3). Spaghetti plots illustrating individual IVM measurements for each animal at the Control, SBG, and Adjacent levels over time are provided in Online Resource 9.

**Table 1:** Median intervertebral motion measured as total rotation (TR) at the synthetic bone graft (SBG), Control, and Adjacent levels preoperatively and at 9 and 12 months after surgery. The SBG vs Control paired difference is presented as the Hodges–Lehmann estimate with corresponding 96.9% confidence intervals (CI), and statistical comparisons were performed using the Wilcoxon matched-pairs signed-rank test. A sensitivity analysis, including the excluded preoperative examination (N = 6), was also performed. Pre-op, preoperatively; M, months after surgery.

Time		Control	SBG	Adjacent segment	Hodges–Lehmann paired difference (96.9% CI), °	P value
		Median (range), °	Median (range), °	Median (range), °		
**Pre-op**	(N = 5)	3.8 (1.2–4.5)	2.1 (1–2.9)	2.6 (2.0–6.8)	−0.85^b^	0.313
**Sensitivity analysis**	(N = 6)	2.8 (0.4–4.5)	1.6 (0.9–2.9)	2.6 (1.3–6.8)	−0.85 (−3.26 to 1.56)	0.438
**9 M**	(N = 6)	0.8 (0.1–2.7)	0.3 (0.04–1.0)	2.5 (1.1–4.7)	−0.57 (−2.62 to 0.10)	0.063
**12 M**	(N = 6)	1.5 (0.2–5)	0.2 (0.07–0.5)	2.9 (2.0–7.5)	−1.28 (−4.86 to −0.07)	0.031^a^

a statistically significant (p < 0.05).

b Exact CI could not be estimated for the primary preoperative analysis because of the small sample size (N = 5).

The median change in IVM TR from before surgery to 9 months or 12 months after surgery did not significantly differ between the Control and SBG levels (9M SBG: 1.0° [0.7–2.5]; Control: 1.1° [0.65–3.8]; p = >0.99; N = 5 and 12M SBG: 2.0° [0.85–2.4]; Control: 0.33° [−1.5–3.9]; p = 0.63; N = 5).

The baseline measurements of the median IVM TR did not significantly differ among the levels designated as Control, SBG, and Adjacent before surgery (Table 1 and Fig. 3; N = 5). A sensitivity analysis including the excluded animal's preoperative examination (N = 6) at baseline did not alter the primary comparison between the Control and SBG designated levels (N = 5: *p* = 0.44; N = 6: *p* = 0.31; Table 1). The comparison between the SBG-designated and Adjacent designated levels showed a similar effect size (N = 5: −1.90°; N = 6: −1.59°), regardless of the inclusion of the excluded examination, although statistical significance changed (N = 5: *p* = 0.063; N = 6: *p* = 0.031). All SBG- and Control-designated levels (6/6) presented a TR above 1° before surgery, and two out of five (2/5) levels presented a TR between 1° and 2° for both the Control and SBG levels (Fig. 3). No significant difference was observed in the median TR of Adjacent segments between time points throughout the study (Fig. 3).

### 3.3 Functional fusion; intervertebral motion <1 degree

Before surgery, none of the SBG, Control, or Adjacent designated levels showed IVM with a TR below 1°. At 9 months after surgery, five out of six (5/6) SBG levels were classified as fused, with a TR of <1°, and at 12 months after surgery, all (6/6) SBG levels were classified as fused, whereas only three out of six (3/6) Control levels were classified as fused at each time point, with TR values of < 1° (Table 2). There was no significant difference in fusion rates between SBG and Control levels at any time point (exact McNemar test: 9M: *p* = 0.50 and 12M: p = 0.25).

**Table 2:** Functional fusion rates of the Control and synthetic bone graft (SBG) levels. Number of levels classified as fused or not fused using a threshold of TR 1° (TR ≥ 1° = not fused, TR < 1° = fused). Pre-op, preoperatively; M, months after surgery.

Time	Control	SBG
Pre-op	0/5	0/5
9 M	3/6	5/6
12 M	3/6	6/6

## 4 Discussion

CT scans are increasingly recognized as the 'gold standard' for the clinical assessment of pseudarthrosis, with dynamic CT evaluations gaining importance as a clinical tool. A recent systematic review highlighted the absence of standardized imaging criteria and emphasized the need for more objective and reproducible quantitative assessment methods (de Vries et al., 2025). The aim of the current pilot study was to evaluate the effect of a hydroxyapatite-based SBG on cervical intervertebral motion (IVM) measured by CT-RSA, in comparison with that of the control in a porcine model of ACDF.

At 12 months postoperatively, IVM was significantly lower at the SBG levels than at the Control levels. This decrease in IVM at 12 months is consistent with the findings of Östman et al., 2021 (Östman et al., 2021), who reported reduced IVM in SBG levels *ex vivo* at 12 months postoperatively, as evaluated by CT-RSA. However, a slightly different SBG and an *ex vivo* model with a standardized biomechanical load were used, whereas the current study assessed IVM *in vivo* over time. In the current study, 12 months postoperatively, all (6/6) SBG levels and only three out of six (3/6) Control levels showed a TR below 1° at 12 months postoperatively, suggesting a high rate of functional fusion at SBG levels, although not statistically significant. Accordingly, these findings should be considered hypothesis-generating rather than definitive evidence of improved fusion rates. In addition, there is no validated CT-RSA-specific threshold for cervical fusion in the literature. The 1° threshold used in the current study was chosen pragmatically, based on the stringent radiographic flexion–extension criteria reported in the literature (Försth et al., 2018; Gruskay et al., 2014; Hipp et al., 2024). Thus, the 1° threshold should be regarded as exploratory. The clinical relevance of this specific threshold requires validation in clinical studies before CT-RSA-specific diagnostic thresholds can be established in the future. Nevertheless, the tendency of increased functional fusion rates at 12 months observed in the current study is in line with a previous study investigating a similar SBG using the same porcine ACDF model (Östman et al., 2024). They demonstrated increased fusion rates as early as 6 and 9 months postoperatively when bony bridging was assessed using CT (Östman et al., 2024). The difference in fusion timing between the studies may be attributed to the different fusion assessments used and the characteristics of the SBGs used. SBGs with slower resorption rates provide a sustained presence of material over time (Habraken et al., 2016). The current study investigated a calcium phosphate ceramic putty consisting mostly of hydroxyapatite, which is slowly resorbed; therefore, a longer period may be needed to achieve fusion and functional stability (Krticka et al., 2021). Additionally, the use of morphological CT evaluation of fusion can sometimes overestimate fusion rates compared with dynamic evaluations (Park et al., 2015). The current study used dynamic IVM analysis, an objective functional evaluation of segmental stability, as a measure of fusion. Thus, this functional stability may take longer to achieve in comparison to a morphological fusion, but is arguably more clinically relevant. A decrease in or absence of apparent movement between the vertebrae does not always align with true bony union, and some motion might still be present during the early healing phases (Gruskay et al., 2014). However, the present study's significant decrease in IVM at 12-month highlights the possible ability of the material to achieve stable segments over time. Combining IVM assessment with CT-based morphological and histological evaluations would provide valuable confirmation of true biological fusion, allow assessment of the quality of bony bridging, and determine whether morphological and histological fusion correlates with functional stability measured by CT-RSA. Morphological and histological evaluations of the vertebral segments in the current study are ongoing to assess true fusion.

The current study represents a relatively new porcine ACDF model using a novel CT-RSA method to evaluate IVM; consequently, validated reference values and predefined thresholds for adequate cervical flexion-extension are not available. Therefore, the total C2–C7 rotation (TR) was assessed at each examination to verify sufficient spinal provocation for a reliable IVM analysis. No significant difference in TR from C2 to C7 was detected between any of the time points. Recently, Hipp et al. (2024) proposed a minimum of 5° of intervertebral rotation or 10% strain at adjacent levels to ensure adequate provocation during dynamic spinal motion studies. Although these criteria have not been validated for the porcine cervical vertebral column, they may represent a useful quality control approach for future CT-RSA studies, complementing or potentially replacing the assessment of overall C2–C7 motion. In the current study, one animal demonstrated markedly reduced baseline preoperative C2–C7 TR compared with the remaining animals, indicating inadequate cervical flexion–extension during image acquisition at this time point. Therefore, this animal was excluded from the primary baseline analysis. In contrast, the total C2–C7 motion of this animal at the postoperative examinations was comparable to that of the remaining animals; therefore, the animal was included in the analysis at postoperative time points. A sensitivity analysis, including the excluded preoperative examination at baseline, did not alter the primary comparison between the Control- and SBG-designated levels. The comparison between the SBG- and Adjacent-designated levels showed a similarly large effect size regardless of the inclusion of the excluded examination, although statistical significance changed, likely reflecting the influence of the small sample size rather than a meaningful change in IVM. However, this difference may also reflect the normal anatomical and regional variation in cervical IVM, as it has been shown that IVM differs between the upper and lower cervical levels in humans and pigs (Wilke et al., 2011). To minimize this potential confounding factor the Control and SBG treatments were randomly assigned to one upper and one lower cervical level in each animal.

At 9 months postoperatively, no significant decrease in IVM was observed at the SBG levels compared with the Control levels, which may be attributed to the small sample size, slightly lower overall degree of cervical column bending at this time point, and potentially fewer levels achieving full fusion at 9 months after surgery than at 12-months. It is reasonable that levels approaching fusion at 9 months postoperatively exhibit partial bridging, perhaps with only anterior bony bridging or through stabilization by soft tissue callus. Because CT-RSA measures the movement of an entire vertebra in relation to the next vertebra, measuring motion at the anterior versus posterior part of the intervertebral space could assist in detecting partial fusions and diagnosing pseudarthrosis (Hipp et al., 2024). Nevertheless, five out of six (5/6) SBG levels, in comparison with (3/6) Control levels, demonstrated a TR < 1° at 9 months after surgery, suggesting a high rate of functional fusion, although not reaching statistical significance.

Recent clinical evidence has shown that the overall cervical range of motion is largely preserved following single-level ACDF, despite successful fusion of the operated level. However, following 2-level ACDF, radiographic analysis consistently reports decreased overall cervical motion, whereas dynamic analysis reports no change in overall cervical motion compared to the baseline (Saad Berreta et al., 2026), suggesting that conflicting conclusions may reflect differences in what each method captures rather than a true contradiction. This is consistent with the findings of the present study, in which no significant changes in C2–C7 flexion–extension TR were observed between time points, despite reduced motion at the operated levels. The discrepancy within the literature between radiographic and dynamic analyses highlights the need for improved standardized and accurate methods for evaluating cervical motion, but also the need for closer alignment between biomechanical measures and patient-reported outcomes, since it has been shown that pain relief may compensate for minimal biomechanical loss (Lai et al., 2016). However, direct comparison of absolute range of motion is limited as flexion–extension in the present study was induced using a standardized externally applied loading protocol under anesthesia in a porcine pre-clinical model. Clinical studies, on the other hand,assess clinical patients using active maximal cervical flexion–extension. Future pre-clinical studies may benefit from incorporating maximal physiological flexion–extension protocols to further improve the comparisons between porcine and human cervical spine biomechanics.

Our results should be interpreted as preliminary findings in the light of several limitations. First, the limitation of using a non-instrumented female porcine ACDF model. Whereas the porcine animal model offers advantages in terms of size, bone metabolism, and spinal anatomy that mimic human conditions, the direct extrapolation of fusion rates and biomechanical responses to human patients requires careful consideration (Hedlund et al., 2022). Additionally, the lack of surgical stabilization differs from standard clinical ACDF practice in humans and may have influenced both healing time and IVM. The use of female mini-pigs is rationalized due to the handling issues of intact boars, however, the potential effects of the estrous cycle and sex hormones on bone healing cannot be excluded. Additionally, the absence of an autologous bone graft positive control limits direct comparison with the current clinical reference standard. However, as this was an exploratory pilot study designed to compare SBG with an empty control, and because only two cervical levels could be operated while preserving an intermediate untreated level, the inclusion of an autologous control was not feasible. Furthermore, although defects were created using a standardized surgical technique and postoperative measurements demonstrated comparable defect volumes between the groups, minor variations in defect size were unavoidable and may also have contributed to variability in the results. Moreover, although 12 months is a common and robust endpoint for pre-clinical spinal fusion studies (Noordhoek et al., 2019), investigating IVM at earlier time points would further improve the understanding of fusion development at an earlier stage, and longer-term follow-up could provide additional value regarding the occurrence of late-stage complications. Additionally, although CT-RSA offers superior precision and functional assessment compared with traditional methods (Sandberg et al., 2023; Brodén et al., 2021), it involves dedicated software and expert personnel, potentially limiting its widespread accessibility in research and clinical settings. Finally, the small sample size reduced the likelihood of detecting significant differences between the groups. However, the paired study design, in which each animal served as its own internal control with both a Control- and a SBG-treated level, reduced between-animal variability and increased statistical efficiency compared with the independent-group design.

In conclusion, this study suggests that a novel hydroxyapatite-based SBG may reduce IVM at 12 months, as assessed by CT-RSA in a porcine ACDF model, and that CT-RSA may be used for objective quantification of segmental stability. These findings support the investigation of SBGs and the feasibility of CT-RSA for functional fusion assessment in pre-clinical studies. Following clinical validation, CT-RSA may complement conventional imaging by providing objective quantitative measurements of IVM and facilitating the evaluation of novel bone graft materials in cervical fusion.

## Data availability

All data supporting the findings of this study are available in the paper and its Supplementary Information.

## Funding

This study was supported by the 10.13039/100000001Swiss National Science Foundation and Vinnova.

## Declaration of competing interests

The authors declare the following financial interests/personal relationships which may be considered as potential competing interests: Franck Forterre reports financial support was provided by Swiss National Science Foundation. Odd Höglund reports financial support was provided by Sweden's Innovation Agency. Maria Östman reports a relationship with Cavix AB that includes: equity or stocks. Michael Pujari-Palmer reports a relationship with Cavix AB that includes: equity or stocks. Franck Forterre reports a relationship with Cavix AB that includes: equity or stocks. Odd Höglund reports a relationship with Cavix AB that includes: equity or stocks. Olof Sandberg reports a relationship with Sectra AB that includes: employment. Michael Pujari-Palmer has patent licensed to Cavix AB. Håkan Engqvist has patent licensed to Cavix AB. If there are other authors, they declare that they have no known competing financial interests or personal relationships that could have appeared to influence the work reported in this paper.
