# Supplementary material for: A hydroxyapatite-based synthetic bone graft reduces intervertebral motion at 12 months, as measured by CT-RSA, in a porcine model of ACDF
Source: Brain Spine. 2026 Aug 13;6:106284. doi: 10.1016/j.bas.2026.106284 (PMC13507529; doi:10.1016/j.bas.2026.106284)
Supplement: Supplementary file 1 [file mmc1.pdf]

Online Resource 1: Experimental animals

Demographics of the experimental animals in the present study. The values are presented as the mean (standard deviations).

**Experimental animals**	
Animal species (strain):	Pig (Ellegaard Göttingen Minipigs, Denmark)
Gender:	Female
Number of animals (N):	6
Age:	23 months
Body weight at start of study:	50 (4) kg
Body weight increase over the study period:	23.8%
Diet:	Standard mini-pig diet containing 1,01% calcium and 0,59% phosphorous, Special Diets Services, UK and hay
Housing:	Single housed in pens approximately 3-5m^2, within sight, smell and sound of one another
Environment enrichments:	Straw bedding, daily 10-15 min exercise in walkway and socialization with the other study-pigs in walkway through the pen bars

A hydroxyapatite-based synthetic bone graft reduces intervertebral motion at 12 months, as measured by CT-RSA, in a porcine model of ACDF.

Maria Östman, DVM,[a] Peter Försth, PhD, MD,[b] Olof Sandberg, PhD,[c] Odd Höglund, PhD, DVM,[d] Patricia Hedenqvist, PhD, DVM,[d] Håkan Engqvist, PhD,[e] Michael Pujari-Palmer, PhD,[d] and Franck Forterre, PhD, DVM[a]

From the [a]Department of Clinical Veterinary Medicine, Division of Small Animal Surgery, Vetsuisse Faculty, University of Bern, Bern, Switzerland; [b]Department of Surgical Sciences, Division of Orthopedics, Uppsala University, Uppsala, Sweden; [c]Sectra AB; [d]Department of Clinical Sciences, Swedish University of Agricultural Sciences, Uppsala, Sweden; [e]Department of Materials Science and Engineering, Division of Applied Materials Science, Uppsala University, Uppsala, Sweden

Correspondence E-mail: maria.oestman@unib.ch
