# Supplementary material for: A hydroxyapatite-based synthetic bone graft reduces intervertebral motion at 12 months, as measured by CT-RSA, in a porcine model of ACDF
Source: Brain Spine. 2026 Aug 13;6:106284. doi: 10.1016/j.bas.2026.106284 (PMC13507529; doi:10.1016/j.bas.2026.106284)
Supplement: Supplementary file 2 [file mmc2.pdf]

Online Resource 2: Randomization

Randomization of the lower and upper levels of the cervical spine receiving SBG or control:

6 Sets of 2 Unique Numbers per Set Range: From 1 (SBG) to 2 (Control)
First number = upper level, second number = lower level

Set #1

2,1 : Control upper level, SBG lower level

Set #2

1,2 : SBG upper level, control lower level

Set #3

2,1 : Control upper level, SBG lower level

Set #4

1,2 : SBG upper level, control lower level

Set #5

2,1 : Control upper level, SBG lower level

Set #6

1,2 : SBG upper level, control lower level

Urbaniak, G. C., & Plous, S. (2013). Research Randomizer (Version 4.0) [Computer program]. http://www.randomizer.org/

A hydroxyapatite-based synthetic bone graft reduces intervertebral motion at 12 months, as measured by CT-RSA, in a porcine model of ACDF.

Maria Östman, DVM,[a] Peter Försth, PhD, MD,[b] Olof Sandberg, PhD,[c] Odd Höglund, PhD, DVM,[d] Patricia Hedenqvist, PhD, DVM,[d] Håkan Engqvist, PhD,[e] Michael Pujari-Palmer, PhD,[d] and Franck Forterre, PhD, DVM[a]

From the [a]Department of Clinical Veterinary Medicine, Division of Small Animal Surgery, Vetsuisse Faculty, University of Bern, Bern, Switzerland; [b]Department of Surgical Sciences, Division of Orthopedics, Uppsala University, Uppsala, Sweden; [c]Sectra AB; [d]Department of Clinical Sciences, Swedish University of Agricultural Sciences, Uppsala, Sweden; [e]Department of Materials Science and Engineering, Division of Applied Materials Science, Uppsala University, Uppsala, Sweden

Correspondence E-mail: maria.oestman@unib.ch
