# Supplementary material for: A hydroxyapatite-based synthetic bone graft reduces intervertebral motion at 12 months, as measured by CT-RSA, in a porcine model of ACDF
Source: Brain Spine. 2026 Aug 13;6:106284. doi: 10.1016/j.bas.2026.106284 (PMC13507529; doi:10.1016/j.bas.2026.106284)
Supplement: Supplementary file 3 [file mmc3.pdf]

Online Resource 3: Anesthesia, analgesia and euthanasia used for experimental animals

Use	Drug (trade name), dose, administration	Manufacturer
Surgical Induction:	Dexmedetomidine, (Dexdomitor), 0,025 mg/kg IM	Orion, Finland
	Zolazepam/Tiletamine (Zoletil), Zolazepam 2,5 mg/kg, Tiletamine 2,5 mg/kg IM	Virbac, Switzerland
	Butorphanol (Dolorex), 10mg/ml, 0,1 mg/kg IM	Intervet, Netherlands
Surgical Maintenance:	Sevoflurane (SevoFlo), 100%, inhelative	Zoetis, Finland
Peri-operative analgesia:	Dexmedetomidine (Dexdomitor), 4 mg/kg/h CRI	Orion, Finland
	or fentanyl (Fentadon), 7mcg/kg/h CRI	Dechra, Netherlands
Postoperative analgesia 7 days:	Buprenorphine (Temgesic) 0,03mg/kg IM	Indivior, Switzerland
Postoperative analgesia 7 days:	Meloxicam (Metacam), 0,6-0,7 mg/kg PO	Boehringer Ingelheim, Germany
Sedation at CT scans:	Zolazepam/Tiletamine (Zoletil), Zolazepam 2,5 mg/kg, Tiletamine 2,5 mg/kg IM	Virbac, Switzerland
	Butorphanol (Dolorex), 10mg/ml, 0,1 mg/kg IM	Intervet, Netherlands
Euthanasia:	Pentobarbital (Euthanimal Vet), 200mg/ml, 100mg/kg IC	VM Pharma, Netherlands

A hydroxyapatite-based synthetic bone graft reduces intervertebral motion at 12 months, as measured by CT-RSA, in a porcine model of ACDF.

Maria Östman, DVM,[a] Peter Försth, PhD, MD,[b] Olof Sandberg, PhD,[c] Odd Höglund, PhD, DVM,[d] Patricia Hedenqvist, PhD, DVM,[d] Håkan Engqvist, PhD,[e] Michael Pujari-Palmer, PhD,[d] and Franck Forterre, PhD, DVM[a]

From the [a]Department of Clinical Veterinary Medicine, Division of Small Animal Surgery, Vetsuisse Faculty, University of Bern, Bern, Switzerland; [b]Department of Surgical Sciences, Division of Orthopedics, Uppsala University, Uppsala, Sweden; [c]Sectra AB; [d]Department of Clinical Sciences, Swedish University of Agricultural Sciences, Uppsala, Sweden; [e]Department of Materials Science and Engineering, Division of Applied Materials Science, Uppsala University, Uppsala, Sweden

Correspondence E-mail: maria.oestman@unib.ch
