# Supplementary material for: A hydroxyapatite-based synthetic bone graft reduces intervertebral motion at 12 months, as measured by CT-RSA, in a porcine model of ACDF
Source: Brain Spine. 2026 Aug 13;6:106284. doi: 10.1016/j.bas.2026.106284 (PMC13507529; doi:10.1016/j.bas.2026.106284)
Supplement: Supplementary file 4 [file mmc4.pdf]

Online Resource 4: Postoperative checklist

Performed every 3 hrs (Q3) first 72h and then 2x/day (Q12) the remaining 7 days.

Pig: _________

Status

	8.00	11.0	14.00	17.00	20.00	23.00	04.00
BR							
Mentation							
Menace							

Posture (in box):

	8.00	11.0	14.00	17.00	20.00	23.00	04.00
Laying down	☐	☐	☐	☐	☐	☐	☐
Sitting	☐	☐	☐	☐	☐	☐	☐
Standing	☐	☐	☐	☐	☐	☐	☐
Kyphotic	☐	☐	☐	☐	☐	☐	☐
other							

Ear position (in box):

	8.00	11.0	14.00	17.00	20.00	23.00	04.00
normal	☐	☐	☐	☐	☐	☐	☐
drooping	☐	☐	☐	☐	☐	☐	☐
Placed backwards	☐	☐	☐	☐	☐	☐	☐
other	☐	☐	☐	☐	☐	☐	☐
Notes							

Activity (in box):

	8.00	11.0	14.00	17.00	20.00	23.00	04.00
normal	☐	☐	☐	☐	☐	☐	☐
hyperactive	☐	☐	☐	☐	☐	☐	☐
inactive	☐	☐	☐	☐	☐	☐	☐
Laying down	☐	☐	☐	☐	☐	☐	☐
other							

Fentanyl patch :

	8.00	11.0	14.00	17.00	20.00	23.00	04.00
Still attached	☐	☐	☐	☐	☐	☐	☐
Folded (% still attached)	☐	☐	☐	☐	☐	☐	☐
Detached	☐	☐	☐	☐	☐	☐	☐
Other							
Removed	☐	☐	☐	☐	☐	☐	☐
New patch on	☐	☐	☐	☐	☐	☐	☐

Pain

Fruit and interaction offered to awake animal:

	8.00	11.0	14.00	17.00	20.00	23.00	04.00
Eating the fruit	☐	☐	☐	☐	☐	☐	☐
Hesitate but eats fruit	☐	☐	☐	☐	☐	☐	☐
Decline fruit	☐	☐	☐	☐	☐	☐	☐
other							

Performed 1x/day (Q24), 1 week postoperatively Date: _______________

______________________days post-op

BW:

- ☐ Filmed when walking towards scale, upo on scale and back
- ☐ Photo taken straight in front of the pig on scale

Weight: _________kg

- ☐ Weight recorded at whiteboard in stable

Neurological evaluation (performed when weighing animal):

Movment in walkway to and from the scale:

- ☐ Normal
- ☐ Ataxia (wobbly)
- ☐ Paresis (tetra/para/hemi)
- ☐ Other_______________

Head position when moving:

- ☐ normal headposition
- ☐ headposition lowered
- ☐ other_______________

Balance when walking:

- ☐ normal
- ☐ wobbly thoracic/pelvic limbs
- ☐ having a hard time to walk
- ☐ other________________

Posturala reactions (cross thoracic/pelvic limbs acc. To picture) – **performed if animal allows**

- ☐ normal thoracic/pelvic limbs (animal correct the limbs position)
- ☐ abnormal thoracic/pelvic limbs (animal does NOT correct the limbs position)

Other notes:

For neurology exam use: Nordquist RE, Meijer E, Staay FJ Van Der, Arndt SS. *Pigs as Model Species to Investigate Effects of Early Life Events on Later Behavioral and Neurological Functions*. Second Edition. Elsevier Inc.; 2017. doi:10.1016/B978-0-12-809468-6/00039-5

For pain face use: Göransson, Lina, 2016. Porcine pain face – identifying visible characteristics of pain in pigs. Second cycle, A2E. Uppsala: SLU, Dept. of Clinical Sciences

A hydroxyapatite-based synthetic bone graft reduces intervertebral motion at 12 months, as measured by CT-RSA, in a porcine model of ACDF.

Maria Östman, DVM,[a] Peter Försth, PhD, MD,[b] Olof Sandberg, PhD,[c] Odd Höglund, PhD, DVM,[d] Patricia Hedenqvist, PhD, DVM,[d] Håkan Engqvist, PhD,[e] Michael Pujari-Palmer, PhD,[d] and Franck Forterre, PhD, DVM[a]

From the [a]Department of Clinical Veterinary Medicine, Division of Small Animal Surgery, Vetsuisse Faculty, University of Bern, Bern, Switzerland; [b]Department of Surgical Sciences, Division of Orthopedics, Uppsala University, Uppsala, Sweden; [c]Sectra AB; [d]Department of Clinical Sciences, Swedish University of Agricultural Sciences, Uppsala, Sweden; [e]Department of Materials Science and Engineering, Division of Applied Materials Science, Uppsala University, Uppsala, Sweden

Correspondence E-mail: maria.oestman@unib.ch
