# Supplementary material for: A hydroxyapatite-based synthetic bone graft reduces intervertebral motion at 12 months, as measured by CT-RSA, in a porcine model of ACDF
Source: Brain Spine. 2026 Aug 13;6:106284. doi: 10.1016/j.bas.2026.106284 (PMC13507529; doi:10.1016/j.bas.2026.106284)
Supplement: Supplementary file 5 [file mmc5.pdf]

Online Resource 5: Computed tomography extension and flexion positioning

All animals underwent flexion-extension CT examinations prior to surgery and at nine- and 12-months post-surgery, which ere performed under a sedation. To achieve controlled and reproducible maximal ranges of motion for the cervical spine in sedated animals, specific positions were employed:

- **Extension position:**
  - The animals were placed in a supine position.
  - The neck was extended outside the examination table to the level of the shoulder joint.

- **Flexion position:**
  - Two identically rolled pillows were consistently placed under the animal's head.
  - The pillows were positioned at the level of the top of the head.

Extension position:

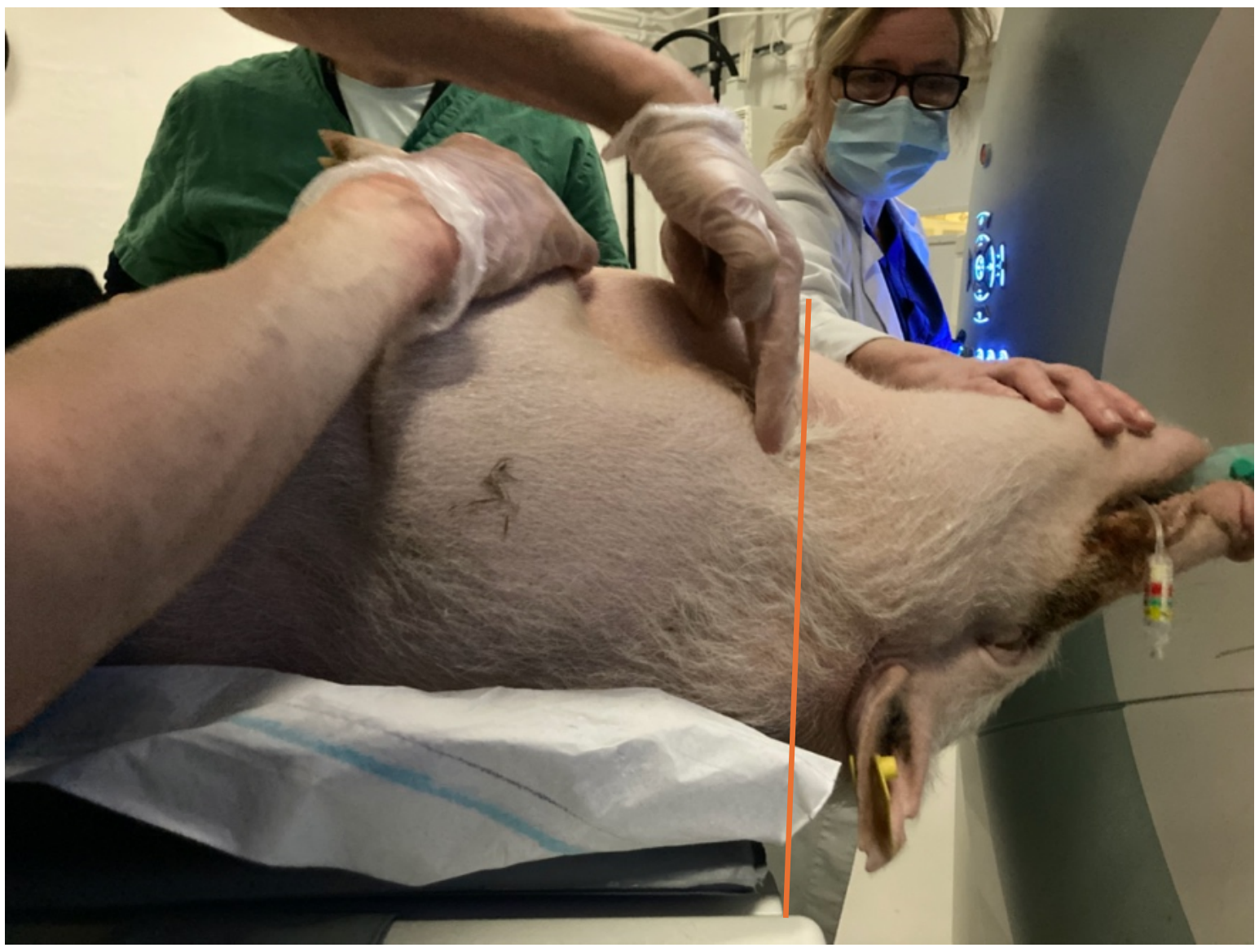

Extension position marked on the CT scan:

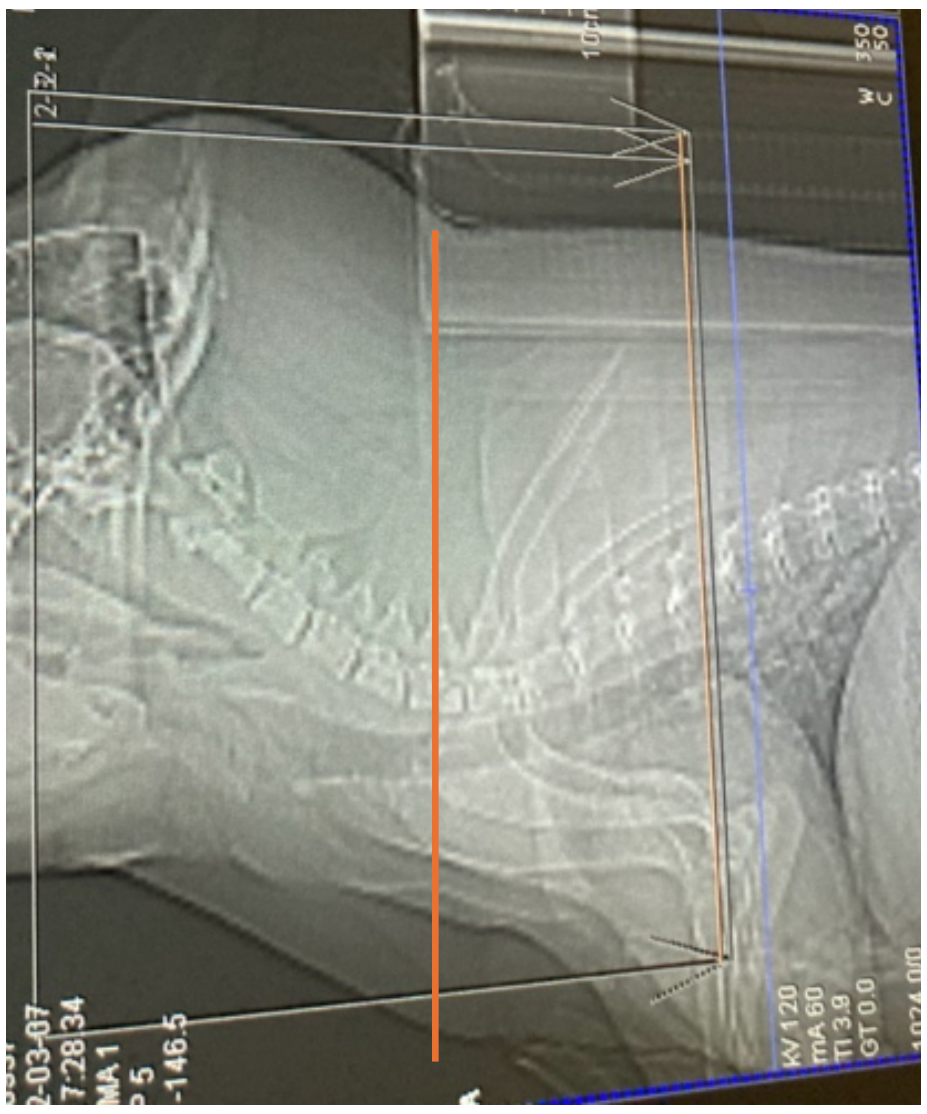

Flexion position side view:

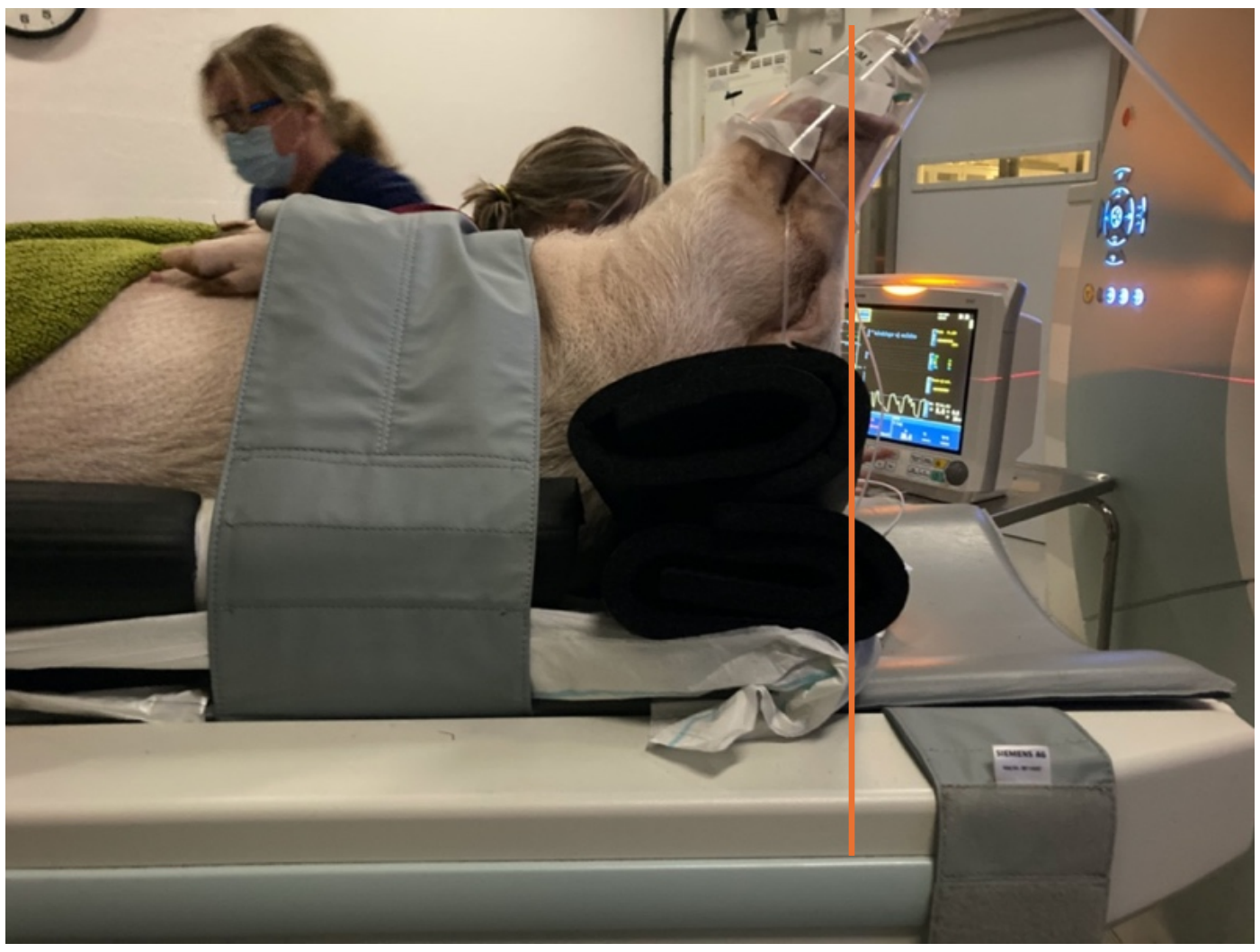

Flexion position front view:

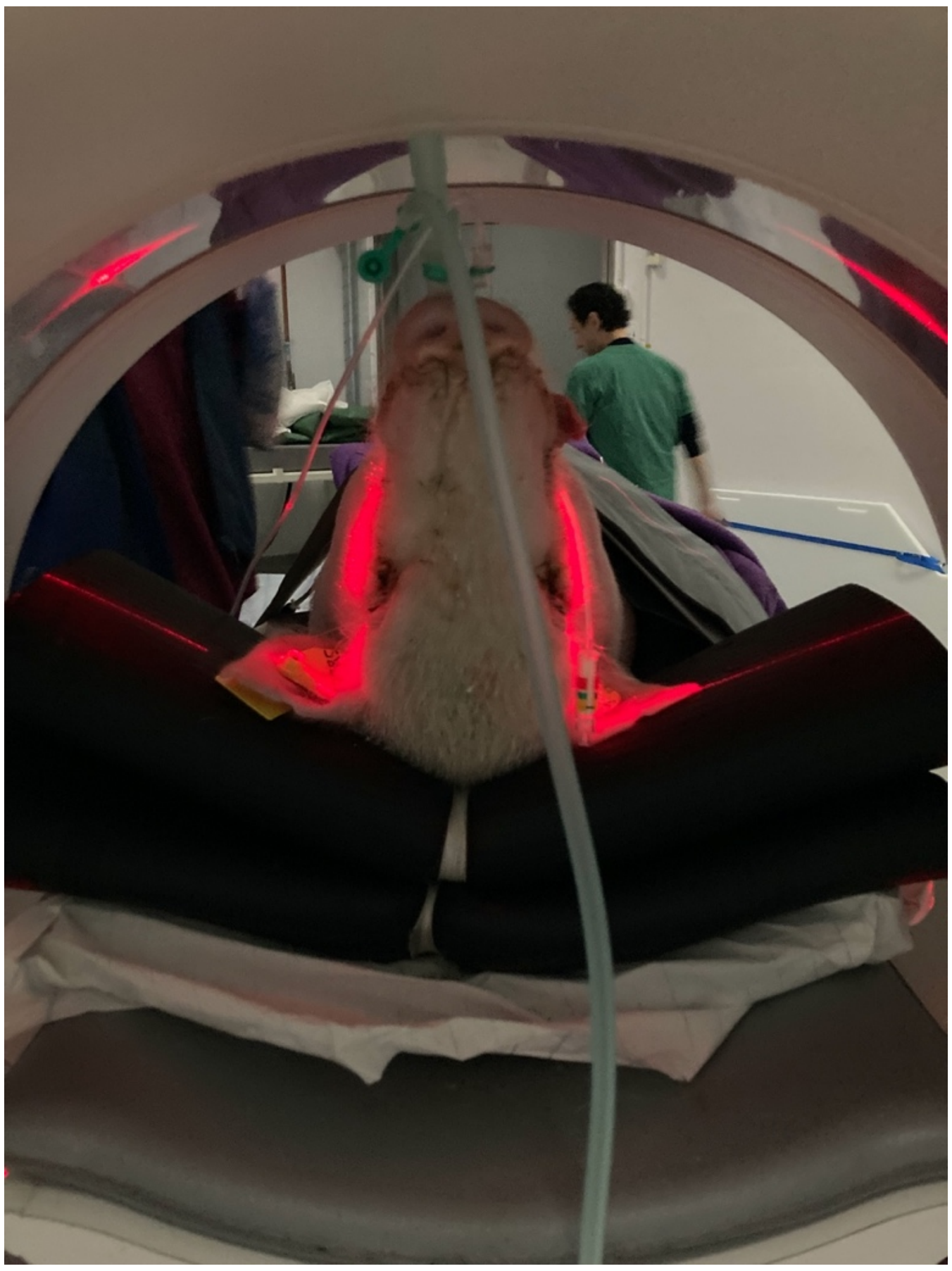

A hydroxyapatite-based synthetic bone graft reduces intervertebral motion at 12 months, as measured by CT-RSA, in a porcine model of ACDF.

Maria Östman, DVM,[a] Peter Försth, PhD, MD,[b] Olof Sandberg, PhD,[c] Odd Höglund, PhD, DVM,[d] Patricia Hedenqvist, PhD, DVM,[d] Håkan Engqvist, PhD,[e] Michael Pujari-Palmer, PhD,[d] and Franck Forterre, PhD, DVM[a]

From the [a]Department of Clinical Veterinary Medicine, Division of Small Animal Surgery, Vetsuisse Faculty, University of Bern, Bern, Switzerland; [b]Department of Surgical Sciences, Division of Orthopedics, Uppsala University, Uppsala, Sweden; [c]Sectra AB; [d]Department of Clinical Sciences, Swedish University of Agricultural Sciences, Uppsala, Sweden; [e]Department of Materials Science and Engineering, Division of Applied Materials Science, Uppsala University, Uppsala, Sweden

Correspondence E-mail: maria.oestman@unib.ch
