# Supplementary material for: A hydroxyapatite-based synthetic bone graft reduces intervertebral motion at 12 months, as measured by CT-RSA, in a porcine model of ACDF
Source: Brain Spine. 2026 Aug 13;6:106284. doi: 10.1016/j.bas.2026.106284 (PMC13507529; doi:10.1016/j.bas.2026.106284)
Supplement: Supplementary file 6 [file mmc7.pdf]

Online Resource 7: CT-RSA protocol

Analysis details:

- 400 HU small smooth with clipboxing
- Extension is primary, flexion is secondary
- Start with scans from 12 months after surgery, then 9 months, then pre-op
- Start with C2-C7, then lower level fused, then upper level fused, then adjacent level.
- The adjacent level is defined as: the level below the level with the least movement at 12 months
- Start with measuring points: proc, then body, then L.fac, then R.fac

Measuring points 1, 2, 3 and 4

Measurepoint 1: Proc;

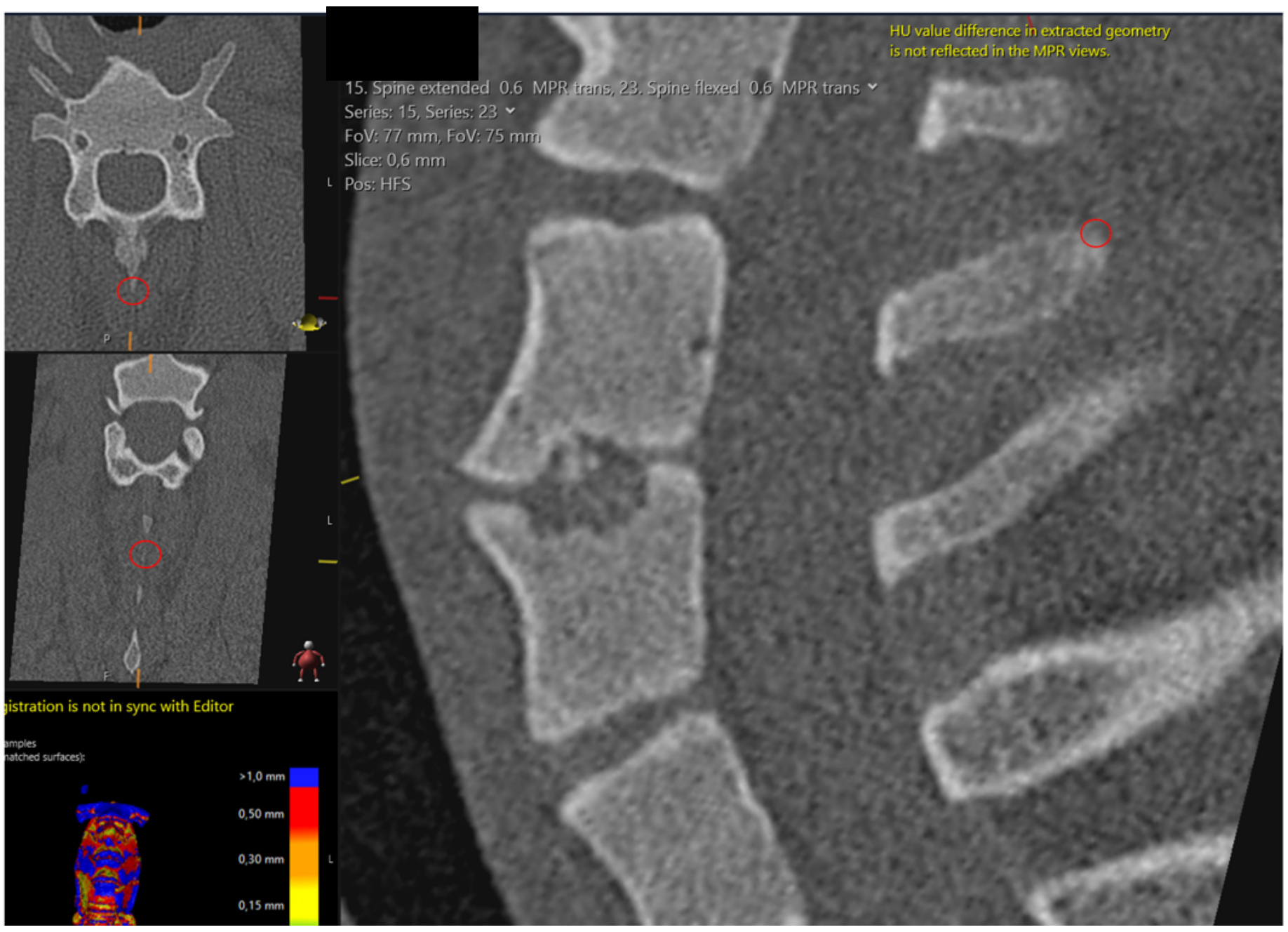

Measurepoint 2: Body;

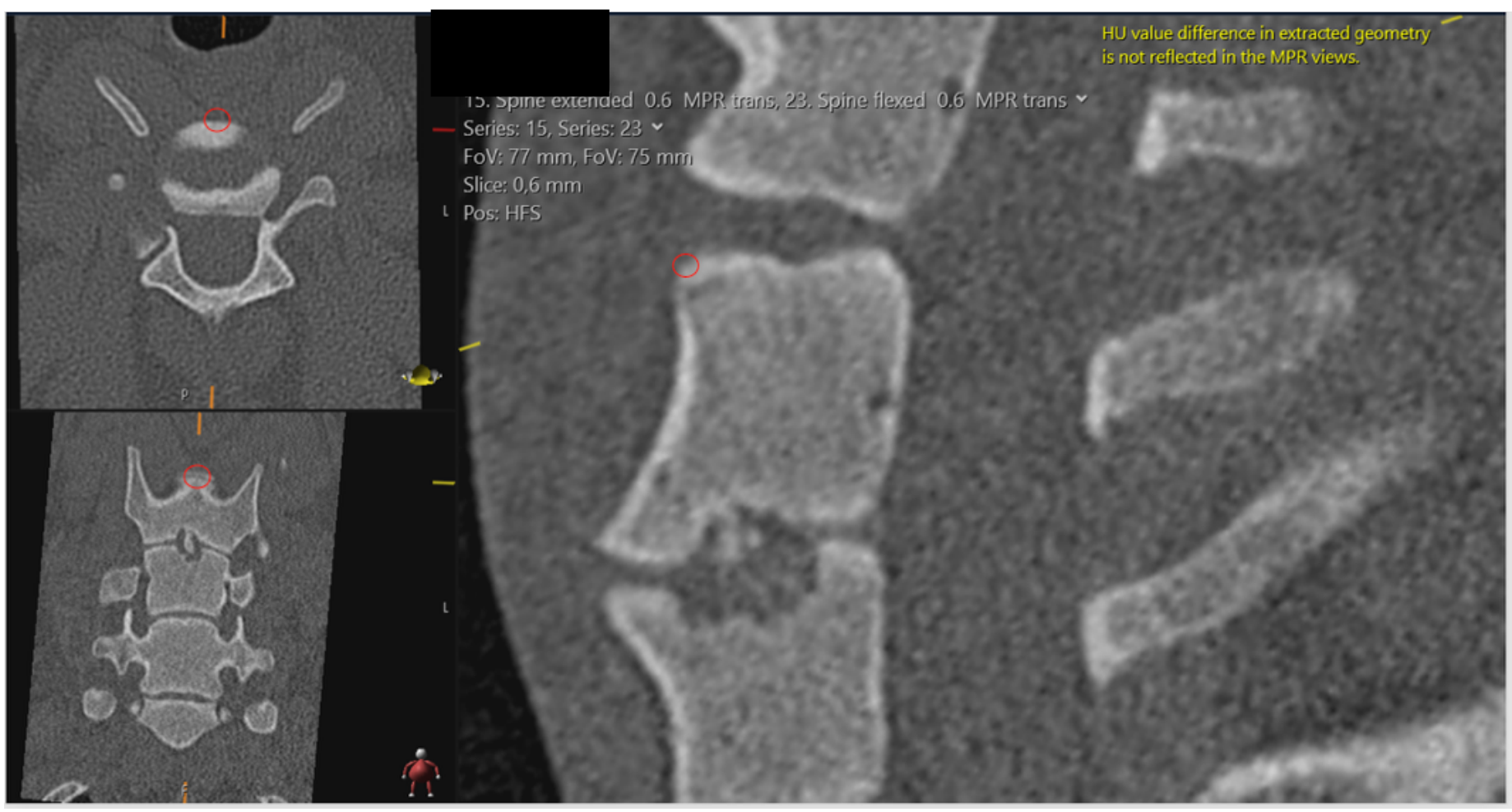

Measurepoint 3 and 4: L.fac. and R.fac;

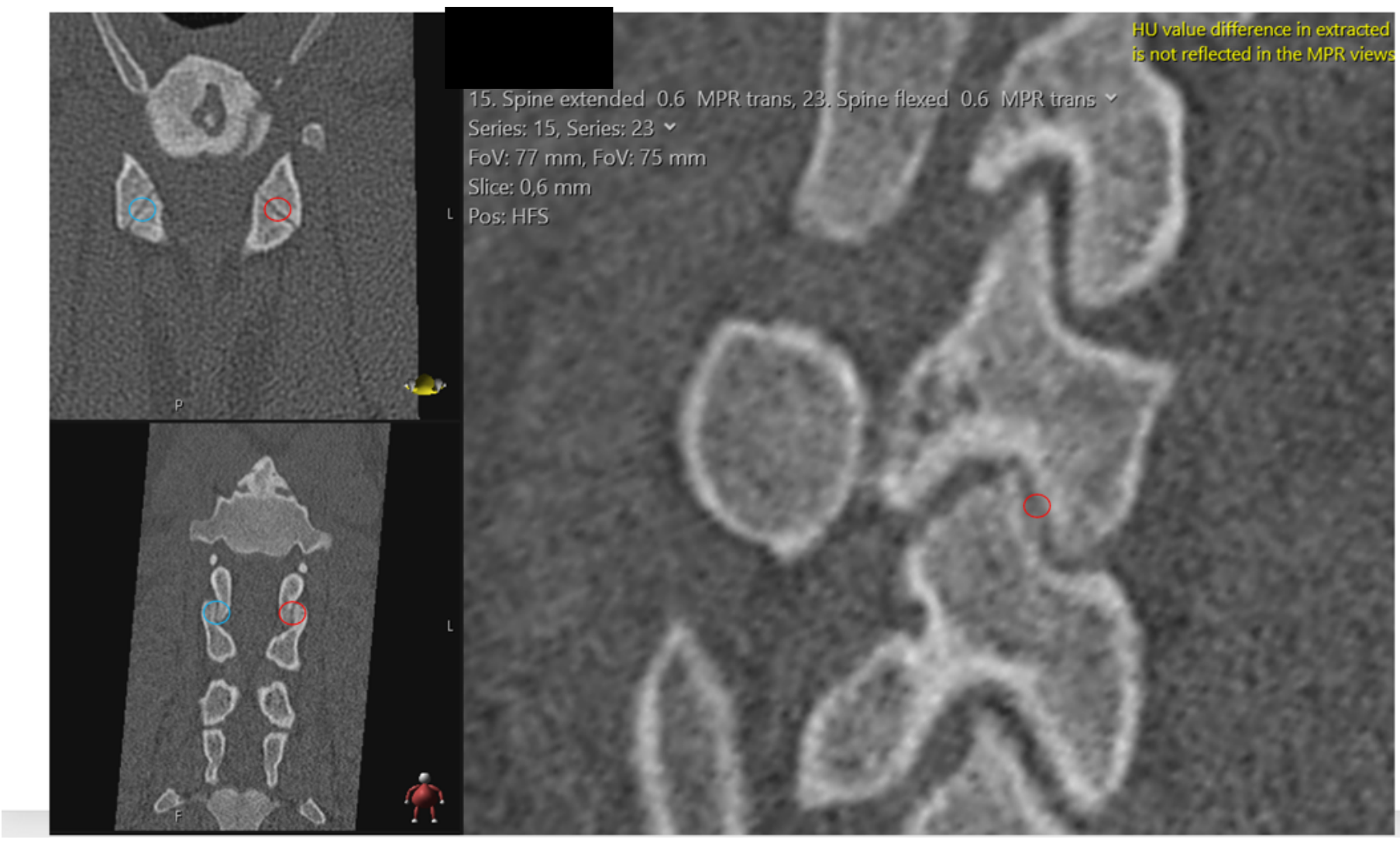

Coordinate-system:

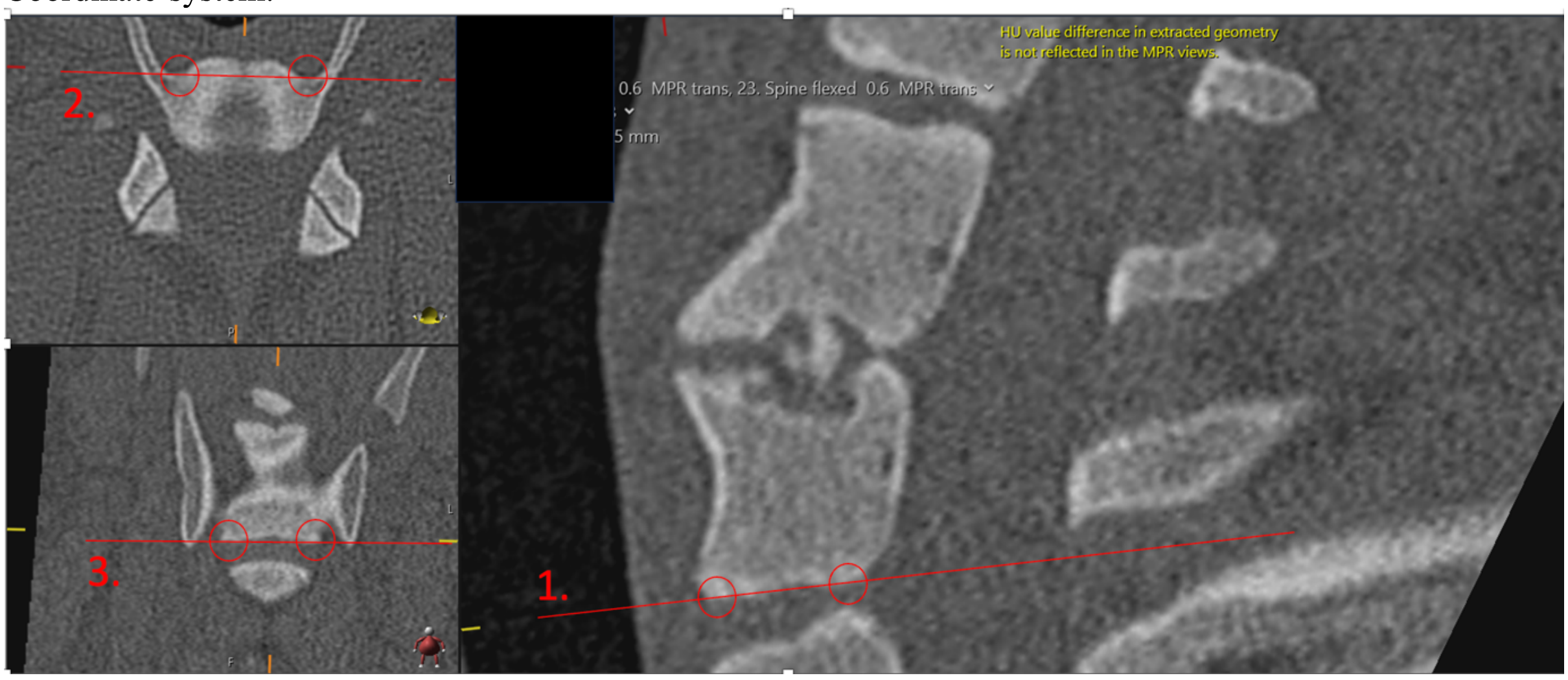

A A hydroxyapatite-based synthetic bone graft reduces intervertebral motion at 12 months, as measured by CT-RSA, in a porcine model of ACDF.

Maria Östman, DVM,[a] Peter Försth, PhD, MD,[b] Olof Sandberg, PhD,[c] Odd Höglund, PhD, DVM,[d] Patricia Hedenqvist, PhD, DVM,[d] Håkan Engqvist, PhD,[e] Michael Pujari-Palmer, PhD,[d] and Franck Forterre, PhD, DVM[a]

From the [a]Department of Clinical Veterinary Medicine, Division of Small Animal Surgery, Vetsuisse Faculty, University of Bern, Bern, Switzerland; [b]Department of Surgical Sciences, Division of Orthopedics, Uppsala University, Uppsala, Sweden; [c]Sectra AB; [d]Department of Clinical Sciences, Swedish University of Agricultural Sciences, Uppsala, Sweden; [e]Department of Materials Science and Engineering, Division of Applied Materials Science, Uppsala University, Uppsala, Sweden

Correspondence E-mail: maria.oestman@unib.ch
