# Supplementary material for: A hydroxyapatite-based synthetic bone graft reduces intervertebral motion at 12 months, as measured by CT-RSA, in a porcine model of ACDF
Source: Brain Spine. 2026 Aug 13;6:106284. doi: 10.1016/j.bas.2026.106284 (PMC13507529; doi:10.1016/j.bas.2026.106284)
Supplement: Supplementary file 7 [file mmc8.pdf]

				Rotations			
ID	Time	Name level	Level	XR	YR	ZR	TR
001	12m	C2-C7	C2-C7	17,415	-0,189	-0,189	17,417
001	12m	Lower fusion	C5-C6	5,03	-0,31	-0,073	5,04
001	12m	Upper fusion	C3-C4	0,151	0,09	-0,003	0,177
001	12m	Adjacent level	C4-C5	3,273	0,4	0,056	3,298
001	9m	C2-C7	C2-C7	9,522	-2,397	-1,098	9,902
001	9m	Lower fusion	C5-C6	2,607	-0,537	0,001	2,662
001	9m	Upper fusion	C3-C4	-0,034	0,005	-0,015	0,04
001	9m	Adjacent level	C4-C5	1,382	-0,52	-0,194	1,49
001	pre-op	C2-C7	C2-C7	13,745	-2,181	-0,628	13,942
001	pre-op	Lower fusion	C5-C6	3,564	-1,291	-0,052	3,792
001	pre-op	Upper fusion	C3-C4	1,022	0,036	-0,106	1,028
001	pre-op	Adjacent level	C4-C5	1,999	-0,316	0,118	2,027
002	12m	C2-C7	C2-C7	10,469	-1,486	-0,144	10,577
002	12m	Lower fusion	C5-C6	0,326	0,009	-0,052	0,331
002	12m	Upper fusion	C3-C4	0,814	-0,028	-0,009	0,816
002	12m	Adjacent level	C6-C7	5,757	-0,548	-0,123	5,785
002	9m	C2-C7	C2-C7	7,509	0,869	-0,102	7,559
002	9m	Lower fusion	C5-C6	0,16	0,014	-0,028	0,163
002	9m	Upper fusion	C3-C4	0,496	0,032	0,033	0,497
002	9m	Adjacent level	C6-C7	4,633	-0,64	-0,086	4,678
002	pre-op	C2-C7	C2-C7	11,58	0,187	0,277	11,586
002	pre-op	Lower fusion	C5-C6	2,695	0,208	-0,043	2,704
002	pre-op	Upper fusion	C3-C4	1,143	-0,106	0,043	1,149
002	pre-op	Adjacent level	C6-C7	5,51	-0,086	-0,036	5,511
003	12m	C2-C7	C2-C7	7,988	-2,182	-0,398	8,296
003	12m	Lower fusion	C4-C5	0,116	-0,05	-0,018	0,131
003	12m	Upper fusion	C2-C3	0,038	0,164	0,106	0,202
003	12m	Adjacent level	C5-C6	2,202	-0,953	0,039	2,4
003	9m	C2-C7	C2-C7	8,992	-0,633	0,18	9,015
003	9m	Lower fusion	C4-C5	0,212	-0,052	-0,052	0,224
003	9m	Upper fusion	C2-C3	-0,036	-0,012	0,117	0,125
003	9m	Adjacent level	C5-C6	2,409	-0,232	0,064	2,421
003	pre-op	C2-C7	C2-C7	5,245	-0,206	0,3	5,257
003	pre-op	Lower fusion	C4-C5	0,934	0,038	-0,029	0,935
003	pre-op	Upper fusion	C2-C3	-0,314	-0,084	0,166	0,367
003	pre-op	Adjacent level	C5-C6	1,297	-0,167	0,097	1,311
004	12m	C2-C7	C2-C7	8,031	-4,17	-1,997	9,327
004	12m	Lower fusion	C5-C6	1,723	-1,304	-0,06	2,162
004	12m	Upper fusion	C3-C4	0,013	-0,004	-0,086	0,088
004	12m	Adjacent level	C4-C5	1,739	-1,294	-0,27	2,186
004	9m	C2-C7	C2-C7	12,423	2,362	0,605	12,671
004	9m	Lower fusion	C5-C6	2,152	-0,592	0,155	2,236
004	9m	Upper fusion	C3-C4	0,987	0,33	-0,04	1,042
004	9m	Adjacent level	C4-C5	2,355	0,771	-0,033	2,478
004	pre-op	C2-C7	C2-C7	15,911	-2,917	0,053	16,173
004	pre-op	Lower fusion	C5-C6	4,269	-1,167	0,178	4,428
004	pre-op	Upper fusion	C3-C4	1,858	-0,886	-0,014	2,059
004	pre-op	Adjacent level	C4-C5	2,477	-0,747	-0,032	2,588
005	12m	C2-C7	C2-C7	15,083	1,456	-0,164	15,152

**005**	**12m**	**Lower fusion**	**C5-C6**	0,54	0,103	-0,061	0,554
**005**	**12m**	**Upper fusion**	**C3-C4**	2,594	0,429	-0,116	2,632
**005**	**12m**	**Adjacent level**	**C6-C7**	7,521	0,234	-0,259	7,529
**005**	**9m**	**C2-C7**	**C2-C7**	6,772	2,212	0,23	7,132
**005**	**9m**	**Lower fusion**	**C5-C6**	0,527	0,113	-0,054	0,542
**005**	**9m**	**Upper fusion**	**C3-C4**	0,951	0,583	-0,038	1,116
**005**	**9m**	**Adjacent level**	**C6-C7**	3,907	0,767	-0,095	3,982
**005**	**pre-op**	**C2-C7**	**C2-C7**	14,503	2,341	0,258	14,697
**005**	**pre-op**	**Lower fusion**	**C5-C6**	2,914	-0,439	-0,055	2,948
**005**	**pre-op**	**Upper fusion**	**C3-C4**	1,769	0,612	0,06	1,874
**005**	**pre-op**	**Adjacent level**	**C6-C7**	6,726	0,841	-0,014	6,778
**006**	**12m**	**C2-C7**	**C2-C7**	8,258	1,39	0,1	8,375
**006**	**12m**	**Lower fusion**	**C4-C5**	0,361	0,004	-0,026	0,363
**006**	**12m**	**Upper fusion**	**C2-C3**	-0,03	0,026	0,057	0,069
**006**	**12m**	**Adjacent level**	**C3-C4**	1,674	1,008	0,206	1,966
**006**	**9m**	**C2-C7**	**C2-C7**	6,824	1,309	0,252	6,955
**006**	**9m**	**Lower fusion**	**C4-C5**	0,521	0,071	-0,031	0,526
**006**	**9m**	**Upper fusion**	**C2-C3**	0,327	0,039	0,035	0,331
**006**	**9m**	**Adjacent level**	**C3-C4**	0,978	0,364	0,054	1,045
**006**	**pre-op**	**C2-C7**	**C2-C7**	14,778	1,995	-2,341	15,055
**006**	**pre-op**	**Lower fusion**	**C4-C5**	4,286	0,206	-0,103	4,292
**006**	**pre-op**	**Upper fusion**	**C2-C3**	0,708	0,748	-0,109	1,036
**006**	**pre-op**	**Adjacent level**	**C3-C4**	1,927	1,601	0,229	2,518

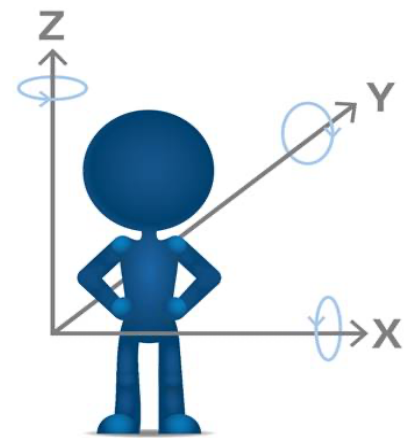

Online Resource 8. Individual CT-RSA-derived intervertebral motion measurements (°).
Raw intervertebral motion measurements for each animal at the Control, synthetic bone graft (SBG), and Adjacent levels before surgery (Pre-op) and at 9 months (9 M) and 12 months (12 M) post-surgery. The Adjacent level was defined as the motion segment immediately below the operated level that demonstrated the least intervertebral motion at 12 months. XR, YR, and ZR represent rotation (°) of the designated moving vertebra relative to the designated rigid vertebra around the X-, Y-, and Z-axes, respectively. TR represents the total rotation (°).

A hydroxyapatite-based synthetic bone graft reduces intervertebral motion at 12 months, as measured by CT-RSA, in a porcine model of ACDF.

Maria Östman, DVM,a Peter Försth, PhD, MD,b Olof Sandberg, PhD,c Odd Höglund, PhD, DVM,d Patricia Hedenqvist, PhD, DVM,d Håkan Engqvist, PhD,e Michael Pujari-Palmer, PhD,d and Franck Forterre, PhD, DVMa

From the aDepartment of Clinical Veterinary Medicine, Division of Small Animal Surgery, Vetsuisse Faculty, University of Bern, Bern, Switzerland; bDepartment of Surgical Sciences, Division of Orthopedics, Uppsala University, Uppsala, Sweden; cSectra AB; dDepartment of Clinical Sciences, Swedish University of Agricultural Sciences, Uppsala, Sweden; eDepartment of Materials Science and Engineering, Division of Applied Materials Science, Uppsala University, Uppsala, Sweden

Correspondence E-mail: maria.oestman@unib.ch
