# Supplementary material for: A hydroxyapatite-based synthetic bone graft reduces intervertebral motion at 12 months, as measured by CT-RSA, in a porcine model of ACDF
Source: Brain Spine. 2026 Aug 13;6:106284. doi: 10.1016/j.bas.2026.106284 (PMC13507529; doi:10.1016/j.bas.2026.106284)
Supplement: Supplementary file 9 [file mmc6.docx]

**Online Resource 6: CT-RSA extension/flexion video**

Computed tomography volumes processed with CT-RSA illustrate intervertebral movement in a typical example from one animal. Illustrating the SBG-treated level (A) and the control level (B) in both induced extension and flexion, observed 12 months after the surgical procedure. The posterior endplate, identified by a red arrow, represents the vertebra undergoing movement, whereas the dashed red line denotes the rigid vertebral body. The resulting intervertebral movement is measured by CT-RSA.

*A hydroxyapatite-based synthetic bone graft reduces intervertebral motion at 12 months, as measured by CT-RSA, in a porcine model of ACDF.*

Maria Östman, DVM,^a^ Peter Försth, PhD, MD,^b^ Olof Sandberg, PhD,^c^ Odd Höglund, PhD, DVM,^d^ Patricia Hedenqvist, PhD, DVM,^d^ Håkan Engqvist, PhD,^e^ Michael Pujari-Palmer, PhD,^d^ and Franck Forterre, PhD, DVM^a^

From the ^a^Department of Clinical Veterinary Medicine, Division of Small Animal Surgery, Vetsuisse Faculty, University of Bern, Bern, Switzerland; ^b^Department of Surgical Sciences, Division of Orthopedics, Uppsala University, Uppsala, Sweden; ^c^Sectra AB; ^d^Department of Clinical Sciences, Swedish University of Agricultural Sciences, Uppsala, Sweden; ^e^Department of Materials Science and Engineering, Division of Applied Materials Science, Uppsala University, Uppsala, Sweden

Correspondence E-mail: maria.oestman@unib.ch
